# High expression of long non-coding RNA NEAT1 indicates poor prognosis of human cancer

**DOI:** 10.18632/oncotarget.17439

**Published:** 2017-04-26

**Authors:** Jian Fang, Fuhao Qiao, Jingjing Tu, Jinfeng Xu, Fangfang Ding, Yun Liu, Bufugdi Andreas Akuo, Jianpeng Hu, Shihe Shao

**Affiliations:** ^1^ Department of Pathogenic Biology, School of Medicine, Jiangsu University, Zhenjiang, Jiangsu 212013, China; ^2^ Department of Urinary Surgery, Zhenjiang First People's Hospital, Zhenjiang, Jiangsu 212013, China

**Keywords:** NEAT1, cancer, OS, meta-analysis, poor prognosis

## Abstract

The nuclear paraspeckle assembly transcript 1 (NEAT1) is a long non-coding RNA. Many studies have reported that NEAT1 plays critical oncogenic roles and facilitates tumorigenesis of various human cancers. High NEAT1 expression is associated with a poor prognosis in cancer patients. This meta-analysis was conducted to assess the association between NEAT1 levels and survival times of cancer patients. Overall survival (OS) was the primary endpoint. Thirteen publications with 1,496 cancer patients from 5 databases (PubMed, EMBASE, Cochrane Library, Wiley Online Library, and Medline) met the criteria for this meta-analysis. Results of the analysis showed that NEAT1 expression in human cancer was significantly associated with OS (hazard ratio [HR]=1.53, 95% confidence interval [CI]: 1.39–1.68), including digestive system tumor (HR=1.54, 95% CI: 1.37–1.73) and respiratory carcinomas (HR=1.44, 95% CI: 1.11–1.85). The results also indicated that NEAT1 expression was highly associated with tumor size (>3 cm vs. ≤3 cm; odds ratio [OR]=2.51, 95% CI: 1.27–4.99; *p*=0.009), TNM stage (III+IV vs. I+II; OR=4.17, 95% CI: 2.42–7.18; *p*=0.00001), and distant metastasis (OR=2.73, 95% CI: 1.28–5.79; *p*=0.01). However, there was no significant association with differentiation (poor vs. well + moderate, OR=1.45, 95% CI: 0.72–2.91) and lymph node metastasis (OR=1.39, 95% CI: 0.54–3.60). This meta-analysis showed that NEAT1 expression may be a useful biomarker for predicting a poor prognosis in patients with cancer.

## INTRODUCTION

Long non-coding RNA (LncRNA) is a class of non-coding RNA, and has more than 200 nucleotides [1]. Initially, LncRNA was considered as spurious transcriptional noise without biological functions because of its lack of protein-coding function [2]. However, later studies indicated that LncRNA plays an important role in pathophysiological processes such as cancers [3, 4]. In recent years, few LncRNAs, such as AFAPI-ASI, MALAT-1, and UCA1 have been confirmed to play a significant role in cancer progression [5–8]. Therefore, LncRNAs might have complex and extensive functions in carcinogenesis and progression of human cancers [9].

The nuclear paraspeckle assembly transcript 1 (NEAT1) is a newly identified nuclear-restricted LncRNA and is located on chromosome 11 (11q13.1). It is an essential component of nuclear paraspeckles [10, 11], and has been confirmed to overexpress in many human cancers, including prostate, lung, and breast cancers [12–14]. High NEAT1 expression in cancerous tissues was reported to be associated with prognosis and overall survival (OS) in several cancers. However, the effect of NEAT1 on the outcome of cancer patients has been controversial. No meta-analysis has been conducted to date on the correlation of NEAT1 with the survival of cancer patients. NEAT1 might act as a potential diagnostic biomarker and prognostic factor. This meta-analysis is the first to explore the correlation between NEAT1 expression and prognosis of cancer patients.

## RESULTS

### Literature search and description of studies

**Figure 1:**
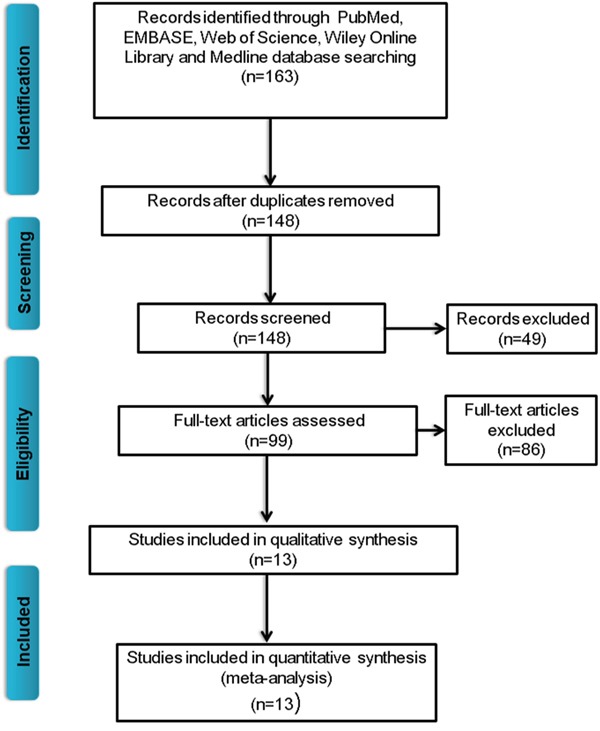
Flow chart of studies selection procedure

As shown on Figure 1, 163 studies were found in the 5 databases (PubMed, EMBASE, Cochrane Library, Wiley Online Library and Medline). We reviewed the titles and abstracts, and 49 irrelevant studies and duplicates were excluded. Of the remaining 99 studies, 86 were eliminated because they used different statistics methods, were animal experiments, or were not in English. After data extraction, 13 studies [14–26], all from China, were selected for the final meta-analysis with a total of 1,496 cancer patients. The main characteristics are summarized in Table 1. The 13 studies covered 9 different types of cancer, including ovarian cancer (n=2), colorectal cancer (n=3), non-small cell lung cancer (NSCLC; n=2), gastric cancer (n=1), hepatocellular carcinoma (n=1), glioma (n=1), nasopharyngeal carcinoma (NPC; n=1), pancreatic cancer (PC; n=1) and esophageal squamous cell carcinoma (n=1). Among the 13 studies, 12 involved tissue collection, and 1 involved a whole blood analysis. All of the detection methods used quantitative polymerase chain reaction.

**Table 1:** Characteristics of studies included in this meta-analysis

Author	Year	Country	Sample size	Sample type	Cancer type	Tumor size (cm)	TNM stage	Follow-up	Method	Outcome	HR statistics	Variance	NOS
						≤3VS>3	I/II Vs III/IV	(Month)				Analysis	
He	2015	China	94	Tissue	Glioma	30/64	23/71	>50	qRT-PCR	OS	Reported	Univariate	8
Guo	2015	China	95	Tissue	HCC	NA	22/73	>60	qRT-PCR	OS	Survival curve	Univariate	7
Fu	2016	China	140	Tissue	GC	NA	63/77	96	qRT-PCR	OS	Reported	Multivariate	8
Sun	2016	China	96	Tissue	NSCLC	41/55	28/68	41	qRT-PCR	OS	Survival curve	Univariate	8
Li	2015	China	239	Tissue	CC	82/157	92/147	>60	qRT-PCR	OS, DFS	Reported	Univariate	8
Wu	2015	China	191	Whole blood	CC	NA	26/165	80	qRT-PCR	OS	Reported	Multivariate	8
Chen	2015	China	96	Tissue	ESCC	NA	35/61	>60	qRT-PCR	OS	Reported	Multivariate	8
Lu	2015	China	71	Tissue	NPC	NA	36/35	>40	qRT-PCR	OS	Reported	Multivariate	8
Aderiaens	2016	Belgium	58	Tissue	OC	NA	NA	>60	qRT-PCR	OS	Reported	Univariate	8
Huang	2016	China	86	Tissue	PC	NA	56/32	>50	qRT-PCR	OS	Survival curve	Univariate	7
Peng	2016	China	56	Tissue	CC	NA	NA	60	qRT-PCR	OS	Survival curve	Multivariate	7
Chen	2016	China	149	Tissue	OC	NA	53/96	>60	qRT-PCR	OS	Reported	Multivariate	8
Pan	2015	China	125	Tissue	NSCLC	60/65	54/71	>40	qRT-PCR	OS	Survival curve	Univariate	8

HCC: hepatocellular carcinoma; GC: gastric cancer; NSCLC: non-small cell lung cancer; CC: colorectal cancer; NPC: nasopharyngeal carcinoma; OC: ovarian cancer; PC: pancreatic cancer; ESCC: esophageal squamous cell carcinoma; NA: not available.

### Correlation of high NEAT1 expression with OS in human cancer

**Figure 2:**
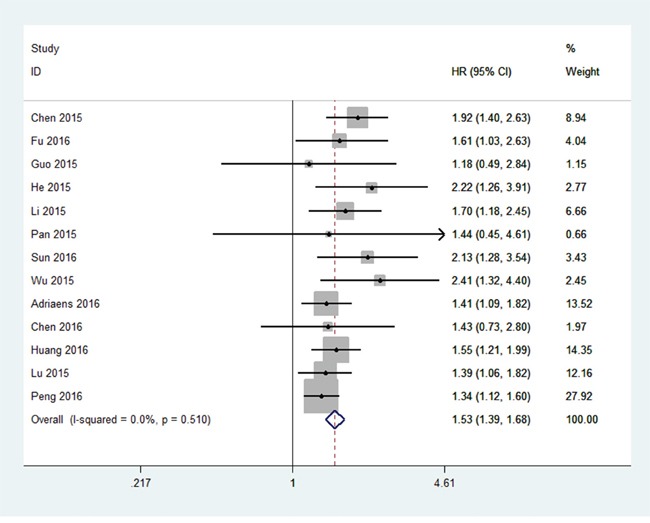
Forest plot of HR for NEAT1 high expression and overall survival

All 13 studies showed OS according to NEAT1 expression levels among the 1,496 patients. The pooled hazard ratio (HR) was 1.53 (95% confidence interval [CI]: 1.39–1.68) for the high NEAT1 expression group versus low expression group (*p*<0.00001; Figure 2). There was no significant heterogeneity (*I^2^*=0%, *P_Q_*=0.51), and the fixed-effects model was chosen to estimate the pooled HR. This suggested that high expression of NEAT1 was a predicator of poor prognosis among human cancers.

### Correlation of NEAT1 expression with clinicopathological parameters and cancer type

**Figure 3:**
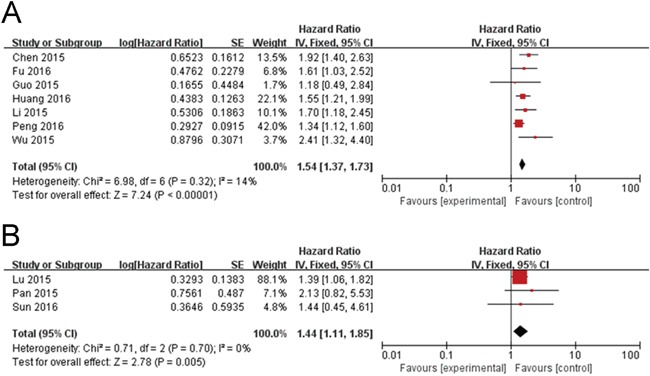
Forest plot of NEAT1 expression with OS in digestive system tumor and respiratory carcinomas patients **(A)** Digestive system tumor and **(B)** respiratory carcinomas.

**Figure 4:**
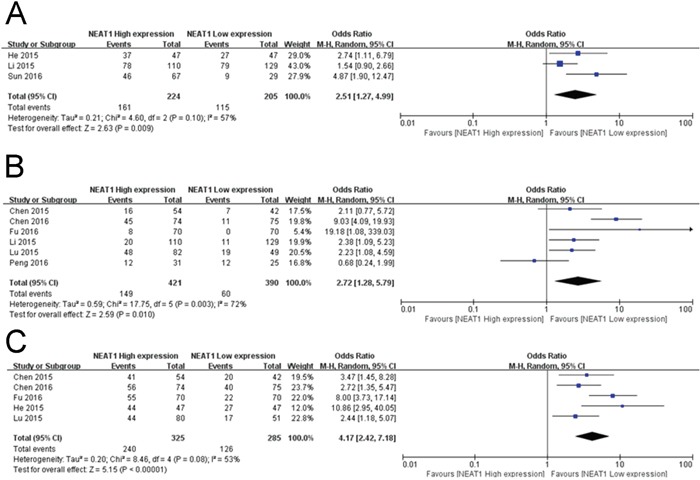
Forest plot of NEAT1 expression and OR for clinicopathological features The investigated clinicopathological parameters are **(A)** tumor size, **(B)** DM and **(C)** TNM stage.

In this meta-analysis, correlation of NEAT1 expression with digestive system tumor and respiratory carcinomas and clinicopathological parameters are illustrated in Figure 3 and Table 2. High NEAT1 expression correlated with poor prognosis of digestive system tumor (HR=1.54, 95% CI: 1.37–1.73; Figure 3A) and Respiratory carcinomas (HR=1.44, 95% CI: 1.11–1.85; Figure 3B) patients. Furthermore, we found correlation of NEAT1 expression with more advanced tumor size (>3 cm vs. ≤3 cm; odds ratio [OR]=2.51, 95% CI: 1.27–4.99; *p*=0.009; Figure 4A), distant metastasis (M1 vs. M0; OR=2.72, 95% CI: 1.28–5.79; *p*=0.010; Figure 4B), and TNM stage (III+IV vs. I+II; OR=4.17, 95% CI: 2.42–7.18; *p*=0.00001; Figure 4C). However, NEAT1 expression was not significantly associated with differentiation (poor vs. well + moderate, OR=1.45, 95% CI: 0.72–2.91; *p*=0.30) and lymph node metastasis (yes vs. no, OR=1.39, 95% CI: 0.54–3.60, *p*=0.5) (Table 2).

**Table 2:** Results of the association between NEAT1 and clinicopathological parameters

Outcome	Studies (n)	OR (HR)	95%CI	P Value	Model	Heterogeneity
						Chi^2^, I^2^, P Value
Tumor size (>3 cm vs. ≤3 cm)	3	2.51	1.27-4.99	0.009	Random	4.60, 57%, 0.10
TNM stage (III+IV vs. I+II)	5	4.17	2.42-7.18	0.00001	Random	8.46, 53%, 0.08
Differentiation (poor vs. well + moderate)	4	1.45	0.72-2.91	0.3	Random	11.97, 75%, 0.007
Lymph node metastasis (Yes vs. No)	5	1.39	0.54-3.60	0.5	Random	29.01, 86%, 0.0001
Distant metastasis (M1 vs. M0)	6	2.72	1.28-5.79	0.01	Random	17.75, 72%, 0.003
Digestive system tumor	7	1.54	1.37-1.73	0.00001	Fixed	6.98, 14%, 0.32
Respiratory carcinomas	3	1.44	1.11-1.85	0.005	Fixed	0.71, 0%, 0.70

### NEAT1 expression in other cancer types

**Figure 5:**
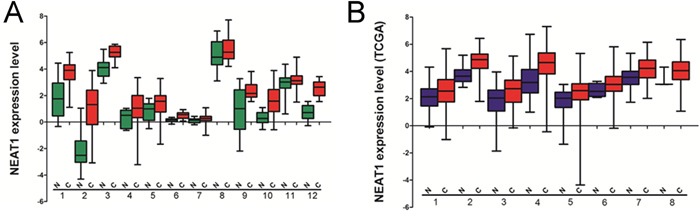
The expression level of NEAT1 analyzed by cancer public database **(A)** The expression of NEAT1 analyzed by Oncomine. 1, prostate cancer; 2, myeloma; 3, breast cancer; 4, lung cancer; 5, gastric cancer; 6, adrenal cancer; 7, colon cancer; 8, liver cancer; 9, renal cancer; 10, lymphoma; 11, pancreas cancer; 12, leukemia. **(B)** The expression of NEAT1 analyzed by TCGA database.1, head and neck squamous cancer; 2, kidney cancer; 3, hepatocellular carcinoma; 4, prostate cancer; 5, stomach adenocarcinoma; 6, uterine corpus endometrioid carcinoma; 7, bladder urothelial carcinoma; 8, cervical squamous cell carcinoma and endocervical adenocarcinoma.

In order to understand the expression level of NEAT1 in other cancer types, we used two public cancer database The Cancer Genome Atlas (TCGA) and Oncomine (https://www.oncomine.org) to analyze the expression of NEAT1 in 15 cancer types. The results indicated that the expression of NEAT1 was higher in the tumor tissues than the corresponding normal issues (Figure 5A and 5B).

### Sensitivity analysis

**Figure 6:**
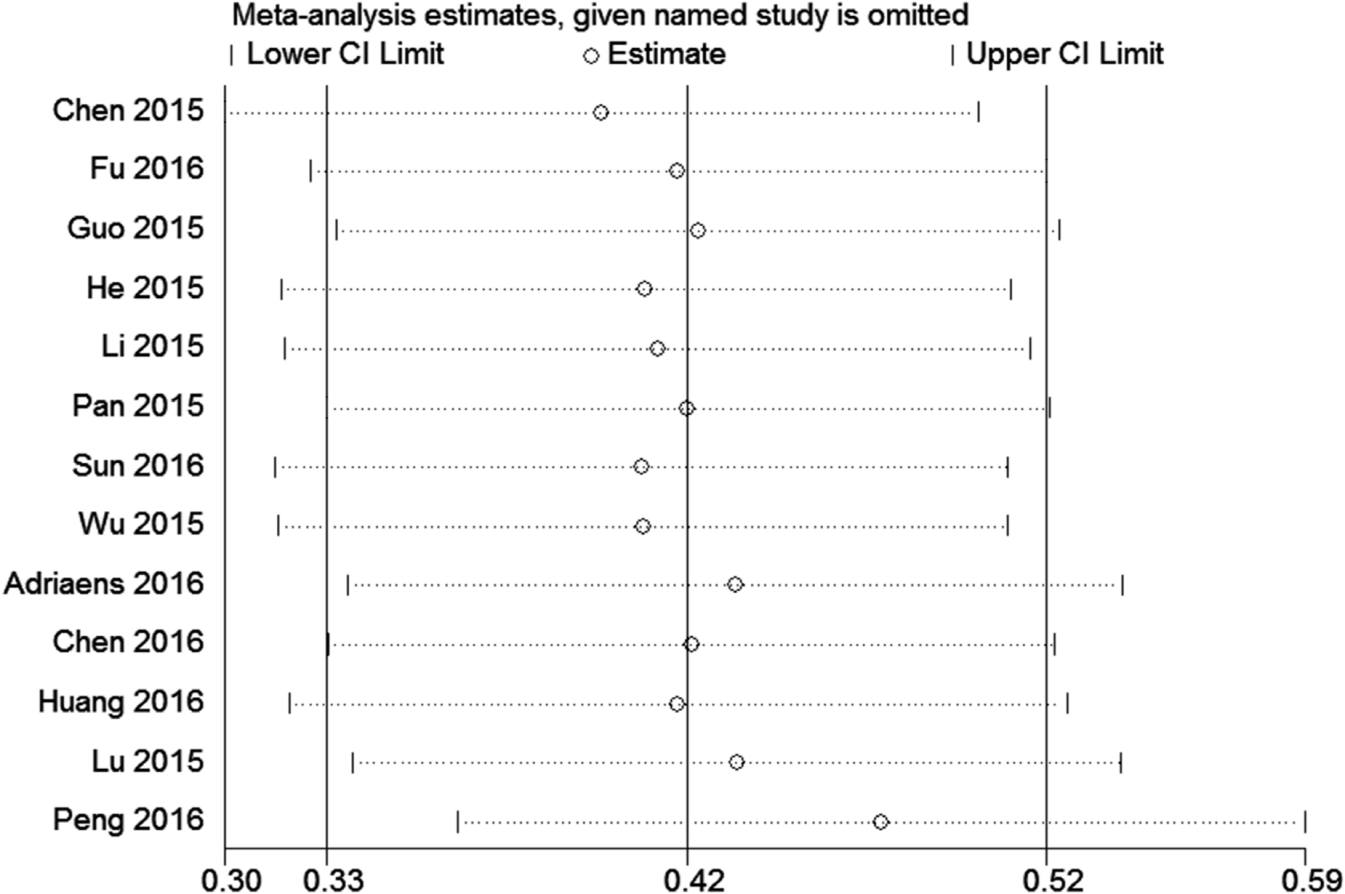
Sensitivity analysis of the effect of the individual study on the pooled HRs for the correlation between NEAT1 expression and overall survival (OS)

A sensitivity analysis was conducted to analyze the association between NEAT1 and OS by deleting one study at a time from the pooled analysis to examine the influence of the removed data set to the overall HR. The result was not influenced by the exclusion of each study, suggesting that the result of the synthetic analysis was robust (Figure 6).

### Publication bias

**Figure 7:**
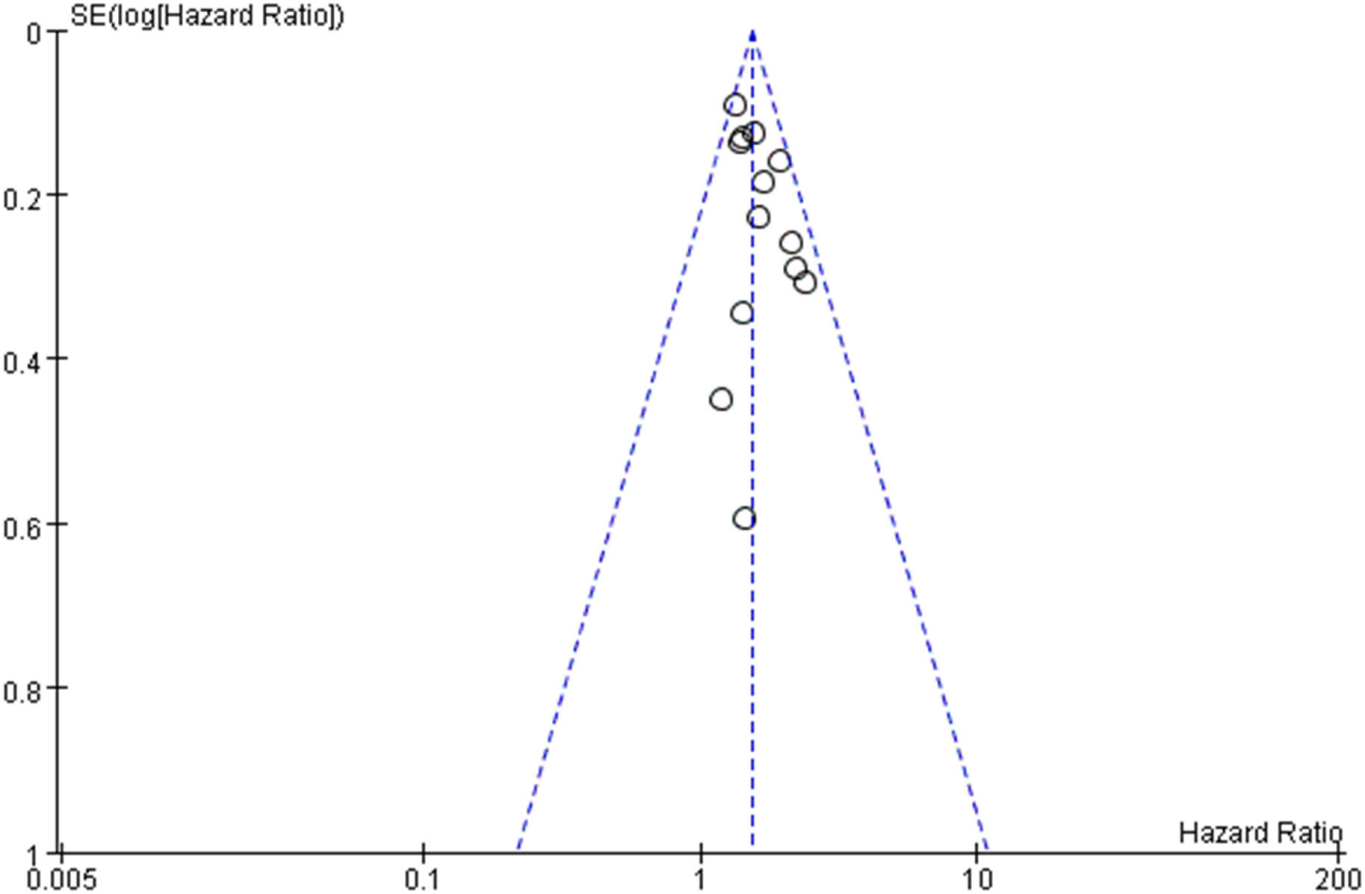
Funnel plot was used to evaluate publication bias on OS

To evaluate the potential for publication bias in this meta-analysis, funnel plots were made (Figure 7). No significant publication bias was observed across studies.

## DISCUSSION

This meta-analysis is the first to evaluate the association between NEAT1 levels and cancer prognosis. The results indicated that high NEAT1 expression was significantly associated with shorter OS times in cancer patients. Subgroup analyses showed that NEAT1 levels were significantly associated with tumor size, TNM stage, and distant metastasis. Yang *et al*.[27] reported that high expression level of NEAT1 was significantly associated with shorter overall survival in cancer patients, which is similar to our results. However, we also found that the expression of NEAT1 was higher in respiratory carcinomas and correlated with poor prognosis of respiratory carcinomas patients. Xiong *et al*.[28] used 57 microarrays and other RNA-seq datasets to analyzed correlation of NEAT1 expression with digestive system tumor, and arrived at the same conclusion with us. These results also shown the reliability of our conclusion.

Recently, many LncRNAs have been reported to function as oncogenes or tumor suppressor genes, including PVT1 [29], MALAT1 [30], and HOTAIR [31]. Choudhry *et al*. reported that NEAT1 is a direct transcription target of HIF-2 in many breast cancer lines and solid tumors. NEAT1 is an essential structural component of paraspeckles, and the hypoxic induction of NEAT1 induces paraspeckle formation in a manner that is dependent upon both NEAT1 and HIF-2. This then leads to accelerated cellular proliferation, improved clonogenic survival, and reduced apoptosis [14]. You *et al*. reported that NEAT1 is a target of miR-449a and is involved in cell growth and apoptosis of lung cancer cell lines [12]. Yoon *et al*. reported that NEAT1 can be modulated by RNA-binding protein AUF1, thus affecting the organization of nuclear paraspeckles [32]. Blume *et al*. reported that NEAT1 is also an important element of the p53-dependent DNA damage response machinery in chronic lymphocytic leukemia [33]. Lastly, Chakravarty *et al*. reported that NEAT1, as an estrogen receptor alpha specific LncRNA, drives oncogenic growth by altering the epigenetic landscape of target gene promoters [13]. Previous studies revealed that NEAT1 overexpression could influence cell apoptosis and migration [34, 35]. On the contrary, silencing of NEAT1 expression by small interfering RNA could suppress cell apoptosis and metastasis. Therefore, NEAT1 expression level may be an indicator of the intrinsic characteristics of cancer progression. Furthermore, the relationship between NEAT1 expression and a variety of cancer patients’ clinicopathological parameters were reported in previous studies [14–21]. These data might explain why high levels of NEAT1 were significantly associated with shorter OS in cancer patients in this meta-analysis. However, the detailed mechanisms of why NEAT1 was associated with oncologic outcome in cancer were unclear. Wu *et al*. reported that a 5-LncRNA signature was detected in clear cell renal cell carcinoma patients and normal controls, and further study indicated that this 5-LncRNA signature appears to provide a promising biomarker for the detection of clear cell renal cell carcinoma [36]. Therefore, NEAT1 might also be a tumor marker for predicting tumorigenesis and cancer progression. Although the results revealed the relationship between NEAT1 and OS and patients’ clinicopathological parameters, there are some limitations of this meta-analysis: the number of studies and cancer patients were limited. Most of the studies were conducted in China; therefore, differences may occur between ethnic groups. Additionally, there were 5 studies in which HR could not directly be calculated, thus they may not have provided the most accurate HR estimate. Our results should be confirmed in further studies.

In all, our results indicated that high NEAT1 expression may be a risk factor for shorter OS and a useful biomarker to predict poorer prognosis in human cancers. However, to reinforce the findings, better standardized methods with large sample sizes are needed to further confirm the association between NEAT1 and clinical outcomes of cancers in various ethnic populations.

## MATERIALS AND METHODS

### Literature search strategies

PubMed, EMBASE, Cochrane Library, Wiley Online Library, and Medline were searched, and articles published from January 1, 1996 to February 10, 2017 were considered. The search strategies were the following: “long non-coding RNA NEAT1” or “lncRNA NEAT1” or “NEAT1” or “nuclear paraspeckle assembly transcript 1” and “Cancer” or “Tumor” or “Neoplasia” and “prognos*” or “surviv*” or “outcome” or “mortality” or “predict.” Reference lists of relevant articles and review papers were also searched manually to identify potential studies.

### Inclusion and exclusion criteria

Inclusion criteria were: (1) Studies were written in English, (2) the level of NEAT1 expression was examined in cancer tissues, (3) investigation of the correlation between NEAT1 expression level and survival outcome (OS or progression-free survival), and (4) HR and its 95% CI for survival time were reported or could be calculated from the reported data. Exclusion criteria were: (1) animal studies, case reports, meta-analyses, and review articles and (2) papers lacking all raw data or inability to calculate its HR, 95% CI, and p values.

### Data extraction and study quality assessment

All data information of eligible studies included the first author's name, publication year, study regions, sample size, cancer type, tumor size, TNM stage, and method of NEAT1 testing. The HR, corresponding 95% CI, and HR statistics for outcome (OS and progression-free survival [PFS]) were calculated independently.

Study quality was assessed using the Newcastle–Ottawa quality assessment scale (NOS). The NOS score items included selection, outcome, and comparability, and ranged between 0 and 9.

### Statistical analysis

The extracted data of this meta-analysis were analyzed using Review Manager 5.3 software (Cochrane network). The HR and its 95% CI were used to evaluate the strength of the association between NEAT1 and OS. If they were reported in a study, HR with 95% CI was extracted directly. If not, the data were extracted from Kaplan–Meier curve using Engauge Digitizer version 4.1 (http://digitizer.sourceforge.net/) [37]. The chi-square-based Q test and *I^2^* statistics were used to determine the heterogeneity [38], and *I^2^* >50% and a *P*-value for *Q* test <0.05 were considered significant heterogeneity. Conversely, the *I^2^*<50% and a *P*-value for *Q* test >0.05 were considered as having no heterogeneity. If there was heterogeneity in the included studies, we chose the random-effects model. The fixed-effects model was chosen when no significant heterogeneity was observed [39]. Sensitivityanalyses were carried out using Stata 12.0 (Stata Corporation, College Station, TX, USA).
